# A Novel Analysis of Compound Muscle Action Potential Scan: Staircase Function Fitting and StairFit Motor Unit Number Estimation

**DOI:** 10.1109/jbhi.2022.3229211

**Published:** 2022-12-14

**Authors:** Maoqi Chen, Zhiyuan Lu, Ya Zong, Xiaoyan Li, Ping Zhou

**Affiliations:** School of Rehabilitation Science and Engineering, University of Health and Rehabilitation Sciences, Qingdao, Shandong 266072, China; School of Rehabilitation Science and Engineering, University of Health and Rehabilitation Sciences, Qingdao, Shandong 266072, China; Department of Rehabilitation Medicine, Ruijin Hospital, Shanghai Jiao Tong University School of Medicine, Shanghai 200025, China; Department of Neurology, Medical College of Wisconsin, Milwaukee, WI 53226 USA; Fischell Department of Bioengineering, University of Maryland at College Park, College Park, MD 20742 USA.; School of Rehabilitation Science and Engineering, University of Health and Rehabilitation Sciences, Qingdao, Shandong 266072, China

**Keywords:** Compound muscle action potential (CMAP), CMAP scan, staircase function, motor unit number estimation (MUNE), StairFit MUNE, spinal cord injury (SCI)

## Abstract

Compound muscle action potential (CMAP) scan provides a detailed stimulus-response curve for examination of neuromuscular disease. The objective of the study is to develop a novel CMAP scan analysis to extract motor unit number estimation (MUNE) and other physiological or diagnostic information. A staircase function was used as the basic mathematical model of the CMAP scan. An optimal staircase function fitting model was estimated for each given number of motor units, and the fitting model with the minimum number of motor units that meets a predefined error requirement was accepted. This yields MUNE as well as the spike amplitude and activation threshold of each motor unit that contributes to the CMAP scan. The significance of the staircase function fit was confirmed using simulated CMAP scans with different motor unit number (20, 50, 100 and 150) and baseline noise (1 *µ*V, 5 *µ*V and 10 *µ*V) inputs, in terms of MUNE performance, repeatability, and the test-retest reliability. For experimental data, the average MUNE of the first dorsal interosseous muscle derived from the staircase function fitting was 57.5 ± 26.9 for the tested spinal cord injury subjects, which was significantly lower than 101.2 ± 16.9, derived from the control group (p *<* 0.001). The staircase function fitting provides an appropriate approach to CMAP scan processing, yielding MUNE and other useful parameters for examination of motor unit loss and muscle fiber reinnervation.

## I. Introduction

Compound muscle action potential (CMAP) scan is an electrophysiological technique that applies hundreds of stimuli to the motor nerve in fine current steps covering subthreshold to supramaximal intensity and records a detailed stimulus-response curve of the examined muscle. CMAP scan can provide comprehensive information on how the CMAP amplitude varies with incremental/decremental stimulus intensities across a full recruitment range of motor units [1], [2]. This is an obvious advantage compared with only sampling a small number of motor units, as typically performed in various motor unit number estimation (MUNE) methods [3], [4], [5]. CMAP scan usually has a sigmoid pattern. The pattern is relatively continuous or smooth for healthy muscles, but shows large steps with significant motor unit loss and muscle fiber reinnervation - a pattern change that can usually be observed with visual inspection.

To characterize CMAP scan, a variety of parameters have been defined such as peak amplitude, S5, S50, S95, step number, step percentage, and number of returners, etc [6], [7]. Delicate processing has been proposed to assess CMAP scan changes, associated with motor unit loss and muscle fiber reinnervation. For example, calculation of “D50” has proved useful in quantifying CMAP scan discontinuities, which is defined as the number of the largest consecutive step differences that builds up 50% of the maximum CMAP [8]. Nandedkar et al. recently reported a CMAP scan index called step index (STEPIX) to reflect changes in the number of motor units [9]. Lu et al. have developed CMAP distribution index (CDIX) to characterize CMAP scan based on calculation of the information entropy [10]. These parameters were tested with CMAP scan data of neuromuscular diseases or injuries and demonstrated expected patterns [8], [9], [10].

Perhaps the most significance of CMAP scan is its contribution to MUNE advancement. Because CMAP scan samples a full recruitment range of motor units, in theory it can overcome biased sample from a small number of motor units, a typical methodology limitation of MUNE. So far two methods have been developed to perform MUNE based on CMAP scan: Bayesian MUNE [11], [12] and MScanFit MUNE [13], [14]. Bayesian MUNE establishes a sophisticated Bayesian model to describe the CMAP scan and estimate the motor unit number. Although the method provides a thorough and comprehensive modeling of the CMAP scan, it is very time-consuming due to the high complexity of the model, and therefore, the method is only used in few research labs. In contrast, MScanFit MUNE is based on a simplified model and quick to implement. MScanFit MUNE compares the recorded CMAP scan with the modeled one, converts it to contour maps, and then uses the degree of overlap of the contours to assess the accuracy of the model. Compared with Bayesian MUNE, the mathematical description of this method is relatively vague, and in practice, the MUNE results can be sensitive to initial settings of the program.

The objective of the current study is to develop a novel analysis of CMAP scan to extract relevant physiological and diagnostic information, including MUNE. Note that in the absence of motor unit alternation and background noise, the CMAP scan will exhibit a strictly increasing staircase pattern. Therefore, the experimental CMAP scan in reality can be viewed as increasing stairs interfered by factors such as motor unit alternation and background noise. Given this basic observation, this study attempts to fit the CMAP scan curve using an increasing staircase function. As a result of searching the best fitting model, MUNE becomes available together with other useful information. The proposed novel analysis was tested using both simulated CMAP scan data and experimental data from healthy control and spinal cord injury (SCI) subjects. The findings indicate that the staircase function fitting provides an appropriate approach to CMAP scan processing, yielding MUNE and other useful parameters for examination of motor unit loss and muscle fiber reinnervation.

## II. Rationale and Algorithms

### A. Problem Description

Generally, curve fitting is performed in the means of least squares. However, due to motor unit alternations and interferences, the CMAP scan curve is not monotonically increasing, which makes the least squares fitting invalid. In this study, a novel method was developed to fit the CMAP scan by an increasing staircase function, which meanwhile provides an estimation of the number of motor units involved in the CMAP scan response.

Let *y*_*t*_ be the measurement of CMAP amplitude recorded at stimulus *x*_*t*_, for t = 1,2, …, N, where *N* is the number of measurements. For convenience we denote the recording amplitudes and stimulus by $\text{y}=\;\left\{y_{1},y_{2},\ldots \;,y_{N}\right\}$ and $\text{x}=\;\left\{x_{1},x_{2},\ldots \;,x_{N}\right\}$, respectively. Here we consider the stimulus intensity to be arranged from small to large, namely $x_{1}\;<\;x_{2\;}<\;\ldots \;<x_{N}$.

Assuming that for each motor unit, it is activated once the stimulus intensity exceeds its threshold and that it makes a stable positive contribution to the magnitude of the CMAP (i.e., all motor units are considered to have consistent positive and negative phases), the CMAP scan curve should ideally be a monotonically increasing staircase function. Consider a CMAP scan model with *M* motor units, which contribute to the CMAP response by two factors: the amplitudes of negative spikes $\mu \;={\left(\mu_{1},\mu_{2},\ldots ,\mu_{M}\right)}^{T}$ and the activation threshold $\tau \;={\left(\tau_{1},\tau_{2},\ldots ,\tau_{M}\right)}^{T}$, where *μ*_*k*_ and *τ*_*k*_ denote the amplitude and activation threshold of the *k*th motor unit. Here, for simplicity we further assume that $\tau_{1}\;<\;\tau_{2\;}<\;\ldots \;<\tau_{M}$. In addition, we give the description of the baseline noise, which is assumed to be normally distributed around its mean *μ*_0_(i.e., the amplitude of the offset), with variance *σ*^2^. For convenience, we define a new variable $\lambda \;={\left(\lambda_{1},\lambda_{2},\ldots ,\lambda_{M+1}\right)}^{T}$. where $\lambda_{i}\;=\;\sum_{k=0}^{i-1}\mu_{k}$, i = 1,2,… ,M +1. **λ** is the cumulative sum of spike amplitudes, i.e., each element in ***λ*** denotes the height of each stair.

Using these notations, we can describe the ideal CMAP scan model in the following concise form:

(1) $$f_{M,\lambda ,\tau }\;(x)=\sum_{i=1}^{M+1}\lambda_{i}I_{\left(\tau_{i-1},\tau_{i}\right]}\left(x\right)$$

where $I_{\Phi }\left(x\right)$ is the indicator function, i.e., $I_{\Phi }\left(x\right)$ takes the value of 1 if x ∈ Φ, otherwise it takes 0. *τ*_0_ and $\tau_{M+1}$ denote the lower bound and upper bound of all the activation thresholds, respectively. Since the distribution range of the activation threshold can be determined before recording the CMAP scan, we might as well further assume that $\tau_{0}\;=\;x_{1}$ and $\tau_{M+1}\;=\;x_{N}$, respectively. Note that in this ideal model, the background noise affects the model only by its offset amplitude *μ*_0_ , without the variance *σ*^2^.

Next, our task is to find the optimal ***λ*** and ***τ*** given the number of motor unit *M* , so that the curve $c_{M,\lambda ,\tau }\left(x\right)\;\;=\;(x,\;f_{M,\lambda ,\tau }\left(x\right))\;$ best matches the CMAP scan curve. This is an optimization problem with 2M + 1 unknown variables. From above analysis, the traditional least squares fitting is not feasible here, and we therefore consider it from a new perspective as described below, where the fitting problem is solved in two steps.

### B. Find the Optimal λ

In order to avoid the influence of motor unit alternation, we only consider the relationship between ***λ*** and **y** first. We hope to find a ***λ*** to best fit **y**, that is, for each *y*_*t*_, the distance to the nearest stair should be as short as possible. To this end, we consider the following optimization problems:

(2) $$\min\;\varphi_{M}\left(\lambda \right)\;=\;\frac{1}{N}\;\sum_{t=1}^{N}\underset{i}{\min}|y_{t}\;-\;\lambda_{i}|\;s.t.\;\;\underset{t}{\min}\;y_{t}\;\leq \;\lambda_{1}\;<\;\lambda_{2\;}\;<\;\cdot \cdot \cdot \;<\;\lambda_{M+1}\leq \underset{t}{\max}\;y_{t}$$

Through further analysis, it can be found that although there are order constraints between the components of ***λ***, the form of the optimization function $\varphi_{M}\left(\lambda \right)$ allows these order constraints to be removed. Then the optimization problem can be simplified as:

(3) $$\min\;\varphi_{M}\left(\lambda \right)\;=\;\frac{1}{N}\;\sum_{t=1}^{N}\underset{i}{\min}|y_{t}\;-\;\lambda_{i}|\;s.t.\;\;\underset{t}{\min}\;y_{t}\;\leq \;\lambda_{1},\;\lambda_{2\;},\ldots \;,\;\;\lambda_{M+1}\leq \underset{t}{\max}\;y_{t}$$

We only need to solve the above problem (3) and sort the results from small to large to obtain the desired optimal solution $\lambda_{opt}^{\left(M\right)}$. Then by using the previously defined relationship between ***μ*** and ***λ***, it is trivial to obtain the optimal amplitudes of negative spikes $\mu_{opt}^{\left(M\right)}$.

This problem can be solved by the generating set search (GSS) method [15]. GSS method is a direct search method, which is particularly suitable for solving the optimization problems of functions that are not differentiable or hard to compute derivatives. It is also worth noting that this problem is very similar to the K-means clustering problem, so the result of K-means clustering can be used as a good initial point of the GSS algorithm.

### C. Solve τ Based on $\lambda_{opt}^{\left(M\right)}$

After obtaining $\lambda_{opt}^{\left(M\right)}$, we further estimate ***τ*** by fitting the CMAP scan curve. To measure the fitness between the estimated staircase curve $c_{M,\lambda_{opt}^{\left(M\right)},\tau }\left(x\right)\;=\;(x,\;f_{M,\lambda_{opt}^{\left(M\right)},\tau }\left(x\right))$ and the CMAP scan curve, we introduce the weighted Manhattan distance. The weighted Manhattan distance between two points $O_{1}\;\left(x_{1},y_{1}\right)$ and $O_{2}\;\left(x_{2},y_{2}\right)$ on the stimulus-amplitude map is defined as $D_{M}(O_{1},O_{2})\;=\;\alpha |x_{1}-\;x_{2}|\;+\;|y_{1}-\;y_{2}|$, where *α* is a weight coefficient, which is taken as 0.1 in this study. The reason for using the weighted Manhattan distance is that we want the optimization process to impose more weights to the gap between the amplitude of observation points and their nearest stairs, so as to reduce the impact of motor unit alternation.

Then the optimal problem can be described as follows:

(4) $$\begin{array}{l} \min\;\phi_{M}\left(\tau \right)\;=\;\;\sum_{t=1}^{N}D\;\left(O_{t},c_{M,\lambda_{opt}^{\left(M\right)},\tau }\left(x\right)\right)\\ s.t.\;\;x_{1}\;<\tau_{1}\;<\tau_{2\;}\;<\;\;\cdot \cdot \cdot \;<\tau_{M}\;<x_{N} \end{array}$$

where $D\left(O,c(x)\right)\;=\;\min_{x}\;D_{M}\left(O,c\left(x\right)\right)$ denotes weighted Manhattan distance between point *O* and curve **c**(*x*), which is defined as the minimum weighted Manhattan distance from point *O* to the points on the curve **c**(*x*). The order constraint can be removed here, so the optimization problem can be simplified to:

(5) $$\min\;\phi_{M}\left(\tau \right)\;=\;\;\sum_{t=1}^{N}D\;\left(O_{t},c_{M,\lambda_{opt}^{\left(M\right)},\tau }\left(x\right)\right)\;s.t.\;\;x_{1}\;<\tau_{1}\;,\tau_{2\;},\;\ldots \;,\tau_{M}\;<x_{N}$$

Similarly, this problem can also be solved by the GSS method and the optimal $\tau_{opt}^{\left(M\right)}$ can be obtained by sorting the optimization results from small to large.

### D. StairFit MUNE

Through the above two-step optimization algorithm, for each *M* , we can estimate the model $f_{M,\lambda_{opt}^{\left(M\right)},\tau_{opt}^{\left(M\right)}}\left(x\right)$ that best fits the CMAP scan. It can be seen that when *M* is large enough, the model can well fit the CMAP scan (in the sense of weighted Manhattan distance). We define that a good fitting is achieved when the averaged error from each point on the CMAP scan to its nearest stair (i.e., $\varphi_{M}$ in Formula (3)) is less than 3 times the standard deviation of baseline noise (i.e., error threshold = 3*σ*). (If the noise is Gaussian distributed, then almost all errors should fall within 3*σ* when the model is well fitted.)

Based on the rule of Occam’s razor [16], we define the minimum *M* that can meet this standard as the estimated number of motor units (StairFit MUNE). It can be seen that the estimation of ***τ*** is indeed not involved in the MUNE estimation. The MUNE becomes available after the first step of optimization to get $\lambda_{opt}^{\left(M\right)}$. The purpose of including ***τ*** in the analysis is to further provide detailed information on motor unit activation threshold.

### E. Algorithm Implementation

#### 1) Parallel Implementation:

The estimation of the best fitting staircase function involves independent calculations for each different motor unit number *M* . Therefore, the parallel computing strategy can be used after defining a reasonable range of motor unit numbers, and the minimum *M* satisfying the error constraint within this range is accepted as the final fitting. Otherwise the program can expand range and continue to search. This parallel strategy can greatly reduce the program running time. In practice, the range of motor unit numbers can be estimated from observing the CMAP scan shape. If obvious steps can be observed, it is more likely associated with a relatively small number of motor units. For a small range, the algorithm can search each *M* in parallel (i.e., resolution 1), while for a large estimated range the search can be performed using odd or even numbers in parallel (i.e., resolution 2) or at higher intervals to increase the efficiency.

#### 2) Error Threshold Setting:

From definition, setting of error threshold depends on estimation of the baseline noise standard deviation *σ* , which becomes the only factor affecting the StairFit MUNE result. Therefore, how to estimate *σ* is very important. If *σ* is overestimated, then MUNE tends to be underestimated; if *σ* is underestimated, then MUNE tends to be overestimated. In theory, *σ* can be estimated by calculating the sample standard deviation of the baseline segment before the minimum activation intensity, while in practice, we found that this approach is not robust for two reasons. First, because of the limited number of samples, the estimated *σ* is very sensitive to interference points. Second, the initial baseline segment (before the minimum activation intensity) is not necessarily an appropriate estimation of the noise level for CMAP data at different intensities. Based on the above considerations, the error threshold for StairFit MUNE was simply fixed to be 15 *μ*V in this study. This implies that the standard deviation *σ* was empirically considered to be 5 *μ*V (given that error threshold = 3*σ*). The rationality of this error threshold setting is demonstrated in the following simulation and experimental studies.

## III. Simulation Study

### A. CMAP Scan Simulation

In the simulation of CMAP scans, a motor unit pool containing a certain number of motor units is simulated first. Each motor unit in the pool is described by a triplet of parameters (μ, τ, ρ). where *μ* and *τ* have the same meaning as above, representing the amplitude and activation threshold, respectively, while *ρ* denotes the relative spread of activation threshold (i.e., the coefficient of variation of the threshold) [17], which is used to simulate the motor unit alternation phenomenon. In addition to this, additive baseline noise is also taken into account to simulate amplitude variations. In this study, *μ* was assumed to follow a two-parameter exponential distribution $\mu \;∼\;E\;(\alpha_{1},\;\beta_{1})$, where *α*_1_ and *β*_1_ are the scale parameter and the location parameter, respectively. This means that the mean and the lower bound of the simulated motor unit magnitudes are $\alpha_{1}\;+\;\beta_{1}$ and *β*_1_, respectively. *α*_1_ and *β*_1_ were set to 200 µV and 25 µV, respectively. The activation threshold *τ* was assumed to follow a gaussian distribution $\tau \;∼\;N\;(\alpha_{2},\;\beta_{2}^{2})$, where the mean threshold *α*_2_ and the standard deviation *β*_2_ were set to 12 mA and 1 mA, respectively. Relative spread *ρ* was considered to follow a uniform distribution $\rho \;∼\;U\;(\alpha_{3},\;\beta_{3})$, where $\alpha_{3}\;=\;0$, $\beta_{3}\;=\;0$. The baseline noise was assumed to be normally distributed around its mean *μ*_0_ , with variance *σ*^2^, where the offset *μ*_0_ was fixed at 10 *μ*V.

After simulating the motor unit pool, electrical stimulus intensities were set to be evenly distributed within a 0.5 mA interval outside the simulated activation threshold distribution to obtain simulated CMAP scan. In other words, the stimulus intensity was uniformly increased from min(*τ* )−0.5 mA to max(*τ* ) + 0.5 mA. The number of stimuli was set to 500.

CMAP scan curves for different situations were simulated. In order to consider the influence of baseline noise on the proposed method, *σ* was set to three levels, namely 1 *μ*V, 5 *μ*V and 10 *μ*V. In addition, the number of motor units *M* was set to 20, 50, 100 and 150, respectively, to test the performance of the proposed method under different numbers of motor units. Therefore, combining *M* and *σ* together, a total of 12 conditions were simulated. For each condition, five trials of simulations were performed. Thus, a dataset of 60 CMAP scans was simulated for performance evaluation (4 motor unit numbers × 3 SNR levels × 5 trials).

### B. Repeatability Testing

Repeatability testing was performed to evaluate the stability of the proposed method. To perform the test, the proposed algorithm was run twice independently on each trial of the simulation data. The results are presented in Table I. For each trial or each CMAP scan data, the average of the results of two independent runs was taken as the final MUNE (not shown in the table for brevity). The averaged MUNE for each condition was calculated as the mean of 5 trials, which is presented in the penultimate column (mean ± SD). It can be observed that MUNE result was not dramatically influenced when *σ* was either underestimated or overestimated, suggesting robustness of the setting of *σ*. The simulation tests were performed on an Intel(R) Core (TM) i7–10750H 2.60 GHz CPU with 16 GB of RAM, and the running time was also reported in Table I (the last column). Note that the running time reported here included searching time for both ***λ*** and ***τ*** . In program running, a parallel computing strategy was used with the resolution set as 1 for 20 motor units, 2 for all the other motor unit numbers.

Relative error of the repeatability was calculated to measure the stability and reproducibility of the MUNE results, which is defined as the percentage of the absolute difference between two MUNEs of a trial to the true number of motor units. The relative errors of repeatability in different conditions are shown in Table II. The average relative error of two independent runs was within 5% under each condition, indicating the stable performance of the proposed algorithm.

### C. Reliability Testing

For the same muscle, it is expected that the MUNE results obtained from the two CMAP scans collected in a time interval should be very close. To this end, a simulated test-retest was implemented. The 60 trials (each corresponding to a different motor unit pool) of the simulated CMAP scans in the repeatability test were viewed as a “test” data. For each trial (or motor unit pool) in the test group, we simulated another CMAP scan as “retest” data using the same condition (i.e., the same motor unit pool and noise level). Then the MUNE result of the retest group was compared with the first result of the test group for reliability testing.

**Fig. 1.**
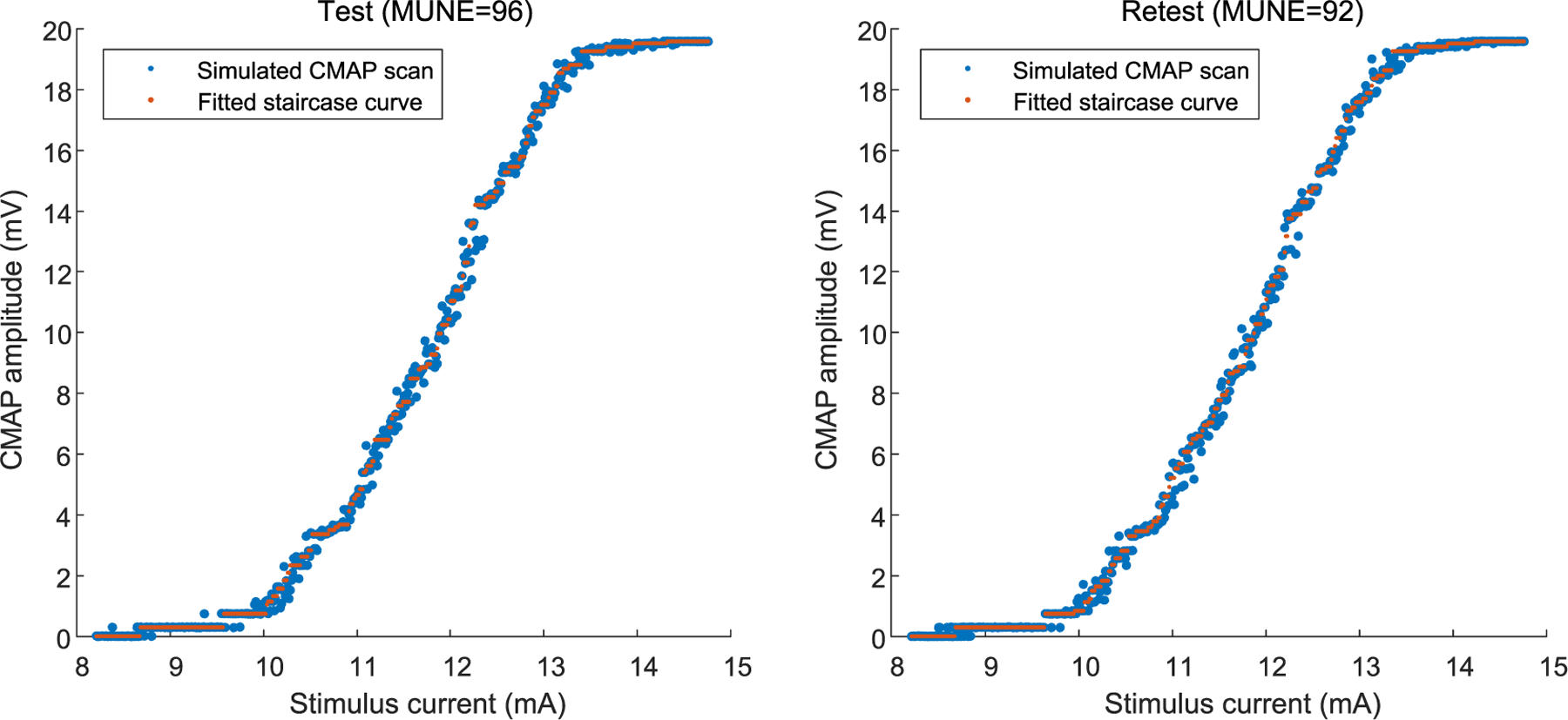
An example of test-retest performance of staircase function fitting of a simulated CMAP scan (motor unit number =100, *σ*= 5 *μ*V).

All the test-retest results are presented in Table III. The averaged MUNE for each condition was calculated as the mean of all the MUNE results (including test and retest) under this condition, which is presented in the last column (mean ± SD). Once again, it can be observed that MUNE result was not dramatically influenced by an under- or overestimated *σ*. Similarly, relative error of reliability is defined as the percentage of the absolute difference between the test and retest MUNE results to the true number of motor units. The relative errors of reliability in different conditions are shown in Table IV. The test-retest reliability (i.e., the Pearson correlation coefficient of two groups) was calculated to be 0.9973. Fig. 1 shows an example of the simulated test-retest CMAP scan data of one trial and the staircase function curve fitting results.

## IV. Experimental Study

### A. Experimental Data Description

The CMAP scan data used in this study were collected from the first dorsal interosseous (FDI) muscle of 13 individuals with SCI tetraplegia (10 males and 3 females) and 13 neurologically intact subjects (8 males and 5 females). The tested SCI subjects had a neurological level ranged from C1 to C7, and American Spinal Injury Association (ASIA) Impairment Scale ranged from A to D, and post injury time ranged from 1 to 24 years. The study was approved by the Committee for Protection of Human Subjects (CPHS) at University of Texas Health Science Center at Houston (UTHealth) and TIRR Memorial Hermann Hospital (Houston, TX). All subjects gave written informed consent in accordance with the Declaration of Helsinki. The detailed subject information and experimental procedures can be found in a previous study [18]. Here we only provide a brief description of CMAP scan recording.

The test was performed on the right hand for the SCI subjects and on the dominant hand (one left hand, 12 right hands) for the control group. Each subject was seated comfortably in a chair with shoulder and elbow flexed 90°, and the forearm rested in semi-prone position on a height-adjustable table. The skin of the hand and wrist was cleaned with alcohol pads. Then Ag–AgCl disposable electrodes (10 mm in diameter) were attached, with active electrode placed on the motor point of the FDI muscle, the reference electrode placed on the distal phalanx of thumb, and the ground electrode placed on the dorsal side of the hand, respectively. An illustration figure of the electrode placement can be found in our previous paper [19]. To deliver electrical stimuli to the ulnar nerve, the stimulating electrode was firmly attached to the skin 1–2 cm proximal to the wrist using surgical tapes and coban self-adherent wraps. The two contact surfaces of the stimulating electrode are 9 mm in diameter, and 20 mm apart. The cathode electrode was positioned distally. The upper current intensity (S100, eliciting all motor units) and lower current intensity (S1, eliciting the first motor unit) were first determined. Then, 500 stimuli (monophasic rectangular impulse, duration: 0.1 ms) were applied to the ulnar nerve with a protocol of linear intensity decline from S00 to S0, and a stimulation frequency of 2Hz. The stimulating current intensity range (S0 to S100) was 5.69 ± 2.20 mA (mean ± SD) to 16.00 ± 4.95 mA for the healthy control subjects, and 8.31 ± 2.4 mA to 19.38 ± 6.68 mA for the SCI subjects.

### B. Experimental Results

**Fig. 2.**
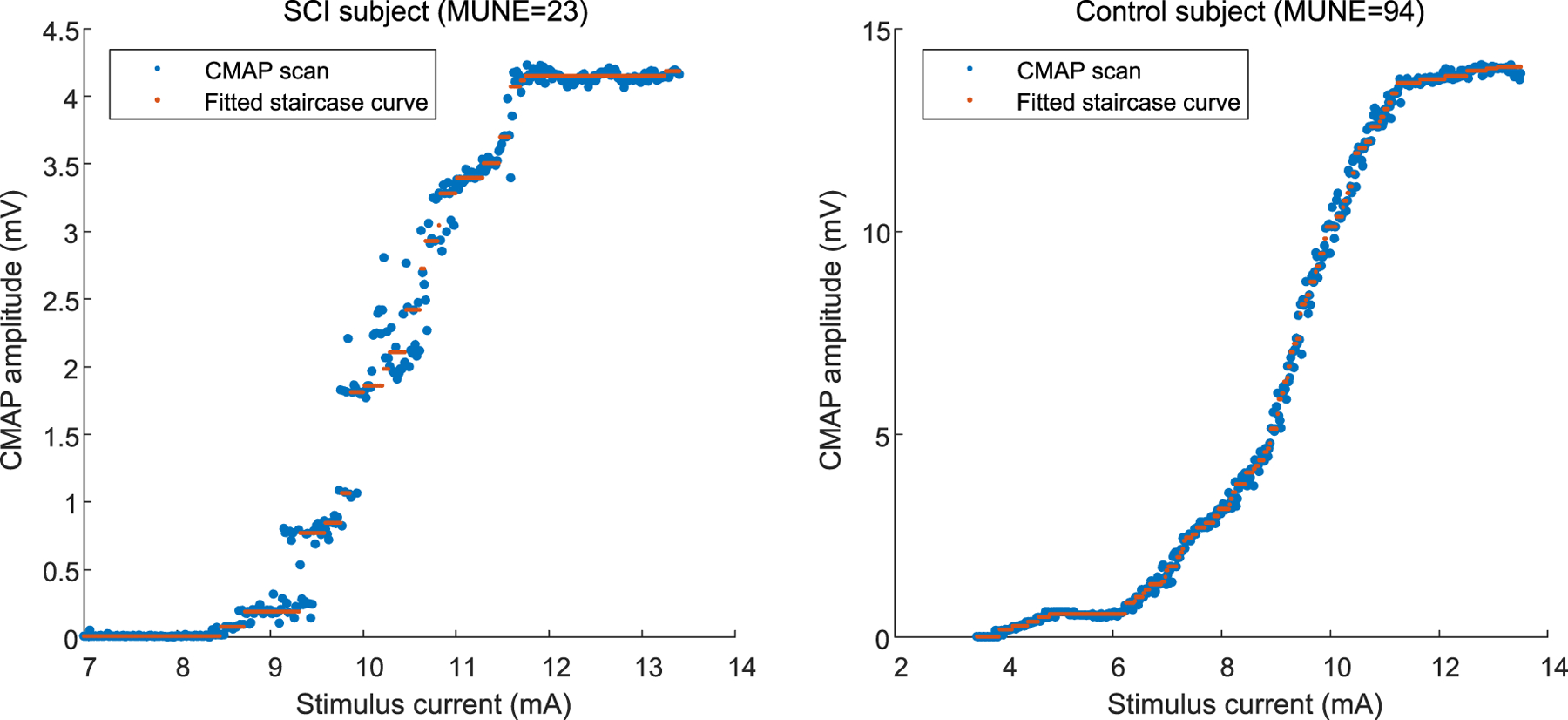
An example of staircase function fitting of experimental CMAP scan data from a representative SCI subject and a healthy control subject.

The staircase function fitting was performed for each recorded CMAP scan. Fig. 2 shows an example of the CMAP scan recorded from a healthy control subject and a SCI subject, respectively. Obvious gaps can be observed in the SCI subject’s CMAP scan, while the control subject showed a relatively smooth pattern. The staircase function fitting is also shown in the figure. It indicates that the motor unit number estimated from the two CMAP scans were dramatically different.

**Fig. 3.**
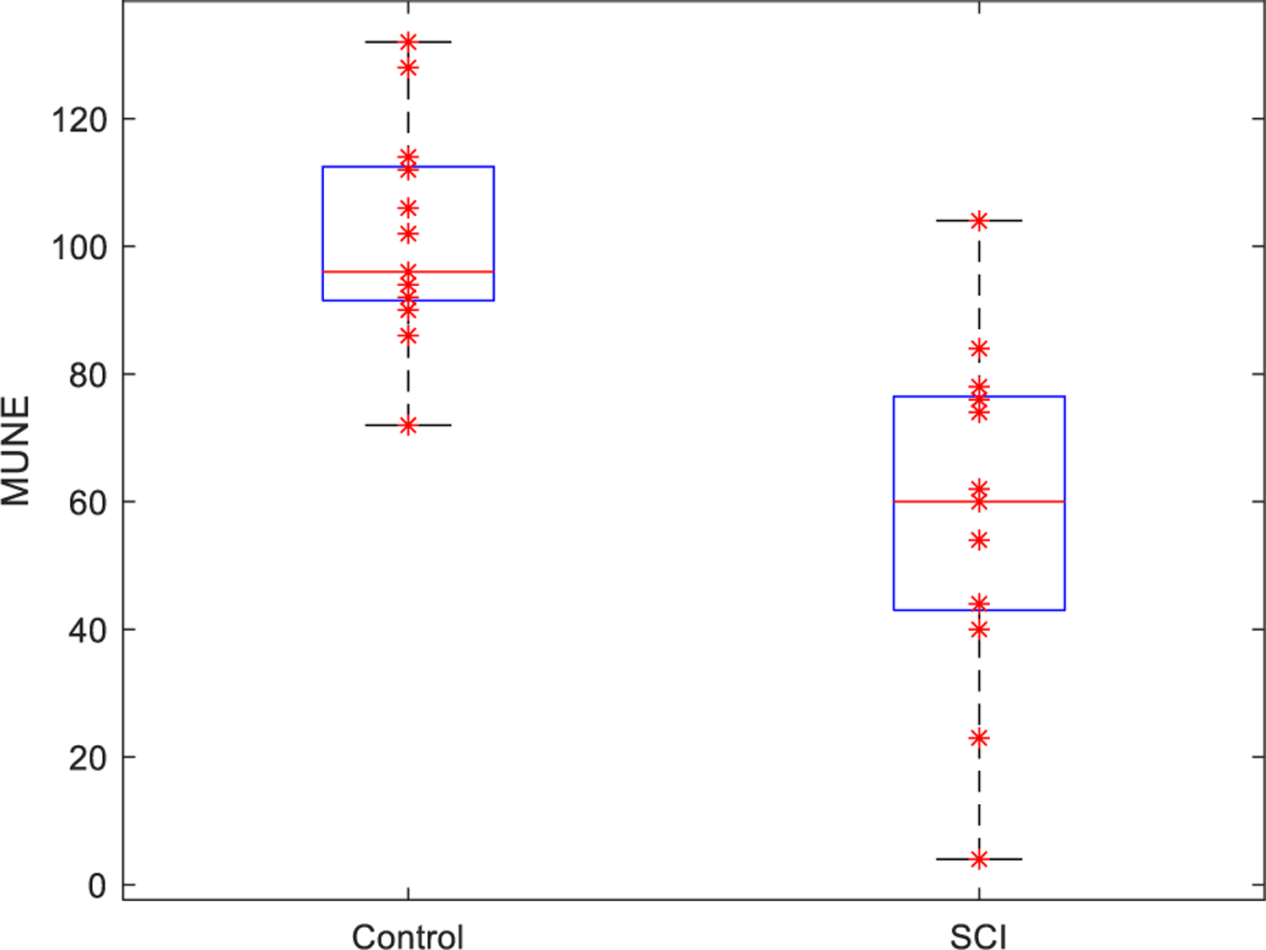
Boxplot of MUNE results of FDI in SCI patient group versus healthy control group.

As shown in Fig. 3, the average MUNE of the FDI muscle derived from the staircase function fitting was 57.5 ± 26.9 for the SCI subjects, which was significantly lower than 101.2 ± 16.9, derived from the control group (p *<* 0.001).

## V. Discussion

### A. Appropriateness of the Fitting Model

This study presents a novel analysis of CMAP scans using staircase function fitting. The ideal CMAP scan curve can be strictly described by a staircase function without considering motor unit alternation and baseline noise. In reality, despite these interference factors, the overall shape of the CMAP scan can still be approximated by a staircase function. Therefore, we chose such a function as the basic mathematical model of the CMAP scan. For each given number of motor units, an optimal staircase function fitting model can be estimated. The fitting model with the minimum number of motor units that meets a predefined error requirement is used as the final fitting model of the CMAP scan. Through such a fitting, the CMAP scan can be studied from a microscopic point of view. The spike amplitude and activation threshold of each motor unit that contributes to the CMAP scan can be estimated, so that the state of the motor units in the muscle can be well simulated to match the experimental CMAP scan.

### B. StairFit MUNE

One important yield of the staircase function fitting of CMAP scan is the estimated motor unit number (StairFit MUNE). Various forms of MUNE methods have been developed since the original incremental stimulation method [20]. In essence, different MUNE methods (such as multipoint or adapted multipoint stimulation MUNE [21], [22], [23], spike triggered averaging MUNE [24], F wave MUNE [25], high density surface EMG MUNE [26], [27]) mainly focus on how to estimate the mean size of single motor unit potentials (SMUP), so that the number of motor units can be estimated by the ratio of CMAP to the mean SMUP. A common problem of previous MUNE methods is their large variance, primarily due to the difficulty in estimating the mean SMUP. Recent advances in MUNE development involve application of CMAP scans, which in nature can overcome the limitation of biased sample from only a small number of motor units. In this sense, the proposed staircase function fitting can be viewed as a novel MUNE method based on CMAP scan, whose performance has been demonstrated by both simulation and experimental approaches in the current study.

### C. Comparison With Bayesian MUNE

The proposed staircase fitting model can be considered as a simplified version of the model in the previous Bayesian MUNE [11], [12]. The Bayesian MUNE method performs a Bayesian approach using Markov chain Monte Carlo to estimate posterior distributions, while the large number of parameters makes the estimation of posterior distributions very time-consuming. Compared with Bayesian MUNE, the proposed staircase fitting estimates a simplified CMAP scan model by solving an optimization problem, which greatly reduces the number of unknown parameters and avoids a large amount of complex calculation work on the estimation of posterior distributions, thus greatly reducing the algorithm complexity. Both methods yield not only the estimated motor unit number, but also other important motor unit characteristics, such as each motor unit’s spike amplitude and activation threshold.

### D. Comparison With MScanFit MUNE

StairFit MUNE also uses a different strategy from MScanFit MUNE [13]. MScanFit MUNE uses 11 operations (including adjusting the parameters of each motor unit, splitting or merging motor units, etc.) to refine the CMAP scan model, and selects the model that minimizes the predefined error score to obtain MUNE. Through repeated simulations of 11 random operations, there may be arbitrarily unlimited model implementations that can meet the error score requirement. MScanFit program (and Bayesian MUNE as well) constrains the minimum amplitude so that the model size cannot grow unlimitedly [11], [12], [13], [14]. In practice the MScanFit output can be sensitive to preset parameters such as relative spread, minimum motor unit magnitude, the initial motor unit number, pre- and post-scan selection, etc.

Theoretically, given the number of motor units, the model with the smallest error can also be approximated by an optimization method. This is the strategy used in StairFit. Although the true optimal solution can hardly be obtained due to too many parameters, a sufficiently close approximation can be achieved. Based on the principle of Occam’s razor [16], the proposed staircase fitting method accepts the model with the smallest motor unit number that meets the preset fitting error requirement as the final fitting. In this sense the MUNE (i.e., the smallest motor unit number) can be viewed as an index of model complexity in describing the CMAP scan. This strategy avoids the problem of overfitting (that may produce a large number of fake motor units with small size), although no restriction is set on the minimum motor unit amplitude. StairFit MUNE is sensitive to the error threshold, which is in fact the only user set parameter for staircase fitting. In this study, the error threshold was empirically set as 15 *μ*V. Although this setting demonstrates reasonable robustness, there is still a margin for improvement.

### E. Potential Pitfalls

Reliable MUNE estimation using StairFit or MScanFit (and in fact, almost all other MUNE methods) has the assumption that all motor units are activated simultaneously and each of them can generate an effective gain in CMAP amplitude above the noise level (i.e., an observable positive contribution to the CMAP amplitude). However, this assumption may be compromised when the motor unit number is too high in a muscle, or when the muscle has large or complex muscle innervation zones (which tends to generate polyphasic action potentials and non-synchronization of motor units). This is a situation we need to be aware when applying StairFit MUNE. Of note, both StairFit and MScanFit MUNE methods apply to CMAP scan, which only contains CMAP amplitude information but not waveform morphological information. Incorporating morphological information of action potentials in the model may be promising to further refine or improve the MUNE results.

### F. Future Work

The staircase function fitting of CMAP scan can be performed automatically and quickly, an advantage making it clinically applicable. The data processing of the fitting usually takes several minutes, depending on the number of motor units. The proposed staircase function fitting was validated with simulated CMAP scan data. It was also applied to experimental CMAP scan data from SCI subjects. Varying degrees of motor unit loss after SCI have been reported in previous literature [28], [29], [30], [31]. StairFit MUNE obtained in this study also demonstrated expected patterns associated with motor unit loss and muscle fiber reinnervation changes after SCI. Nonetheless, MUNE has the most applications in amyotrophic lateral sclerosis (ALS) patients. More experimental studies are warranted to further demonstrate the potential and significance of the proposed method for examination of neuromuscular diseases, particularly for tracking disease progression in patients with ALS. It also remains future work to compare the proposed method with other CMAP scan processing methods (such as Bayesian MUNE, MScanFit MUNE, STEPIX, CDIX, D50), to test the proposed method with different muscles, and to examine its sensitivity to different experimental protocols (different step numbers and stimulus pulse widths).

## Figures and Tables

**TABLE I:** Repeatability Testing of STairFit MUNE Using Simulated CMAP Scan Data With Different Motor Unit Number (20, 50, 100, 150) and Noise Level (1 *μ*V, 5 *μ*V, 10 *μ*V) Inputs

#MU	$\sigma \left(\mu V\right)$	MUNE											Time (s)
		Trial 1		Trial 2		Trial 3		Trial 4		Trial 5		Averaged	
		Run 1	Run 2	Run 1	Run 2	Run 1	Run 2	Run 1	Run 2	Run 1	Run 2		
20	1	20	21	19	19	15	14	16	17	17	17	17.5±2.3	12.5±2.0
	5	18	18	19	20	14	15	18	18	18	18	17.6±1.8	
	10	22	22	17	17	17	19	23	26	20	20	20.3±3.0	
50	1	44	46	50	54	44	42	52	54	40	42	46.8±5.4	66.9±7.9
	5	52	56	48	50	52	54	54	56	54	54	53.0±2.3	
	10	56	56	52	52	56	56	70	70	64	66	59.8±7.4	
100	1	104	104	84	84	108	112	94	92	104	106	99.2±10.5	206.9±29.0
	5	104	106	102	104	114	116	96	98	106	106	105.2±6.5	
	10	96	96	92	94	120	120	116	118	102	104	105.8±12.2	
150	1	164	166	126	126	128	130	164	164	134	132	143.4±19.4	432.1±75.1
	5	150	152	156	156	154	154	154	154	132	132	149.4±9.9	
	10	144	142	168	168	136	142	154	154	160	164	153.2±11.7	

**TABLE II:** The Relative Errors of Repeatability of StairFit MUNE in Different Conditions (Motor Unit Number, Noise Level of Cmap Scan Simulation

#MU	$\sigma \left(\mu V\right)$	relative error of repeatability (%)					
		Trial 1	Trail 2	Trail 3	Trail 4	Trail 5	Averaged
20	1	5	0	5	5	0	3.0±2.7
	5	0	5	5	0	0	2.0±2.7
	10	0	0	10	15	0	5.0±7.1
50	1	4	8	4	4	4	4.8±1.8
	5	8	4	4	4	0	4.0±2.8
	10	0	0	0	0	4	0.8±1.8
100	1	0	0	4	2	2	1.6+1.7
	1	0	0	4	2	2	1.6±1.7
	10	0	2	0	2	2	1.2±1.1
150	1	1.3	0	1.3	0	1.3	0.8±0.7
	5	1.3	0	0	0	0	0.3±0.6
	10	1.3	0	4	0	4	1.9±2.0

**TABLE III:** Test-retest Reliability Testing of StairFit MUNE Using Simulated CMAP Scan Data With Different Motor Unit Number (20, 50, 100, 150) and Noise Level (1 *μ*V, 5 *μ*V, 10 *μ*V) Inputs

#MU	$\sigma \left(\mu V\right)$	MUNE										
		Trial 1		Trial 2		Trial 3		Trial 4		Trial 5		Averaged
		Test	Retest	Test	Retest	Test	Retest	Test	Retest	Test	Retest	
20	1	20	19	19	18	15	14	16	19	17	17	17.4±2.0
	5	18	19	19	20	14	14	18	19	18	17	17.6±2.1
	10	22	22	17	18	17	17	23	22	20	20	19.8±2.4
50	1	44	50	50	54	44	46	52	50	40	40	47.0±4.9
	5	52	52	48	46	52	54	54	56	54	54	52.2±3.0
	10	56	58	52	50	56	58	70	68	64	68	60±7.0
100	1	104	106	84	88	108	106	94	96	104	104	99.4±8.4
	5	104	108	102	102	114	108	96	92	106	98	103.0±6.5
	10	96	92	92	96	120	120	116	120	102	98	105.2±12.3
150	1	164	156	126	128	128	132	164	164	134	144	144.0±16.4
	5	150	144	156	152	154	150	154	154	132	128	147.4±9.8
	10	144	132	168	164	136	144	154	154	160	156	151.2±11.8

**TABLE IV:** The Relative Errors of Test-Retest Reliability of StairFit MUNE in Different Conditions (Motor Unit Number, Noise Level) of CMAP Scan Simulation

#MU	$\sigma \left(\mu V\right)$	relative error of reliability (%)					
		Trial 1	Trial 2	Trial 3	Trail 4	Trial 5	Averaged
20	1	5	5	5	15	0	6.5±5.5
	5	5	5	0	5	5	4.0±2.2
	10	0	5	0	5	0	2.0±2.7
50	1	12	8	4	4	0	5.6±4.6
	5	0	4	4	4	0	2.4±2.2
	10	4	4	4	4	8	4.8±1.8
100	1	2	4	2	2	0	2.0±1.4
	5	4	0	6	4	8	4.4±3.0
	10	4	4	0	4	4	3.2±1.8
150	1	5.3	1.3	2.7	0	6.7	3.2±2.8
	5	4	2.7	2.7	0	2.7	2.4±1.5
	10	8	2.7	5.3	0	2.7	3.7±3.0
